# Dysphagia Advances in Head and Neck Cancer

**DOI:** 10.1007/s40136-023-00445-6

**Published:** 2023-02-15

**Authors:** J. M. Patterson, M. Lawton

**Affiliations:** School of Health Sciences, Institute of Population Health/Liverpool Head and Neck Centre, University of Liverpool, 1.17 Thompson Yates Building, The Quadrangle, Brownlow Hill, Liverpool, L69 3GB USA

**Keywords:** Outcome measures, Treatment, Prehabilitation, Speech-language pathology, Deglutition disorders, Head and neck neoplasm

## Abstract

**Purpose of Review:**

This review summarises the current literature regarding head and neck cancer–associated dysphagia. Up-to-date evidence for dysphagia outcome measurement for this population is provided, in addition to recent innovations that aim to prevent, reduce or remediate the common and debilitating side effects of treatment.

**Recent Findings:**

Both patient-reported outcomes and clinical measures are necessary to capture the multi-dimensional nature of swallowing. A minimally important difference in scores has been calculated for some of these measures, to aid interpretation and powering of clinical trials. The number of dysphagia-related trials has increased, predominantly investigating optimal treatment for oropharyngeal HPV-positive disease, and speech and language pathology interventions using an impairment-based approach.

**Summary:**

Although substantial progress has been made, further work is necessary to establish a consensus over outcome measures. Modifying treatments may improve outcomes. Several trials are underway to establish the effectiveness of speech and language pathology dysphagia interventions.

## Introduction

Dysphagia is one of the most common and debilitating consequences of head and neck cancer (HNC) and its treatment, with devastating implications for quality of life and health status. While HNC treatments are continually evolving to minimise side effects, early identification of dysphagia is imperative to prevent potential complications and timely rehabilitation. Swallowing is a multifactorial function, and thus, different perspectives are necessary to obtain a holistic evaluation. In the first section, advancements in swallowing outcome measurement will be considered. Following this, although not an exhaustive review, recent evidence for HNC treatments aiming to reduce dysphagia severity will be summarised, ending with a review of advances in prehabilitation and rehabilitation.

### Dysphagia Outcome Measurement

Outcome measurement is fundamental to research and is integral to good clinical practice. A large number of dysphagia tools are available, some specific to HNC, and are either clinician-rated or patient-reported [1••]. Each offers a unique perspective, with the association between these tools being variable and therefore they are not interchangeable [2]. An overview of commonly employed measures, and where available, their psychometric properties are provided below. In addition, the minimally important difference, i.e. the smallest difference in score which patients perceive as beneficial will be referenced, to enable interpretation by the patient, clinician and researcher.

### PROs

A patient-reported outcome (PRO) is defined as ‘any report of the status of the patient’s health condition that comes directly from the patient, without interpretation of the patient’s response by a clinician or anyone else’ [3]. They are used to collect information about patients’ experiences of symptoms, and their impact on quality of life, enabling individual and group level monitoring and identification of those in need of intervention. Most HNC-specific quality of life questionnaires include swallowing-related domains. These domains are, however, limited in the number of component dysphagia questions. Many dysphagia-specific PROs are available — a systematic review of their psychometric properties for those used in HNC dysphagia is underway [4].

The MD Anderson Dysphagia Inventory (MDADI) is the mostly commonly used dysphagia-specific quality of life PRO for clinical and research purposes and was specifically developed for patients with HNC [5]. It has 20 items, divided into four domains: global (one item), emotional (six items), physical domain (eight items) and functional (five items). It has been translated into numerous languages with subsequent reliability and validity testing. Exploratory factor analysis suggests the MDADI could be abbreviated to just five key questions for clinical purposes, although further validation is required [6]. A between-group minimally clinical important difference of 10 points has been calculated for the total score [7]. It has been used as a primary or co-primary outcome measure for a number of clinical trials of HNC treatment [8–11] and swallowing rehabilitation [12, 13].

The Swallowing Quality-of-Life Questionnaire [14] is a 44- item scale assessing 10 domains: food selection, burden, mental health, social functioning, fear, eating duration, eating desire, communication, sleep and fatigue. The SWAL-QOL has excellent reliability and validity and a minimally important difference of 12 points has been established [15]. It has also been used as a primary outcome measure for clinical trials of HNC rehabilitation [16].

The Eating Assessment Tool (EAT-10) [17] is a list of ten dysphagia symptoms, validated on a general dysphagia population. The EAT-10 has proven reliability and validity; work to establish responsiveness in HNC, a minimally important difference and cut-off score remains under scrutiny [18]. The Swallowing Outcomes After Laryngectomy (SOAL) questionnaire was developed for patients with laryngectomy [19]. Its responsiveness and minimally important difference is untested. The Sydney Swallow Questionnaire was initially validated in patients with neurological disorders and subsequently in patients with HNC [20]. It has been translated into a number of languages. A cutoff point for identifying swallowing dysfunction has been published [21].

### Clinician-Rated Measures

1. Clinical tests

There are a number of simple clinical tests commonly employed as an outcome measure. These are usually quick, cheap to conduct and repeatable, without the need for expensive instrumentation.

Diet restrictions are an important domain to capture, although ratings may be influenced by factors other than oropharyngeal dysphagia such as loss of teeth, odynophagia and dysgeusia. Dietary texture restrictions are usually assessed using clinician-rated scales. The two most commonly used scales are the normalcy of diet (NoD), a sub-section of the Performance Status Scale for HNC [22] and the Functional Oral Intake Scale (FOIS) (originally developed for stroke patients) [23]. The two scales are subtly different; with the NoD recording the most complex texture, the patient can manage using examples of drinks and food, whereas the FOIS scores feeding method (i.e., tube and/or oral) and type of food consistencies. The FOIS has been used as a primary outcome measure for case control and randomised controlled trials (RCT) evaluating swallowing rehabilitation [24, 25]. Elsewhere, duration of feeding tube dependence has been used as a primary outcome to evaluate the timing of swallowing exercises — although is a surrogate measure of oropharyngeal dysphagia [26•].

A more direct assessment of swallowing performance using a clinical test can be achieved using the 100-ml water swallow test. This is a simple, timed test, where the patient is asked to swallow 100-ml water as quickly as is comfortably possible. From this, measures of swallow volume (mls per swallow) and capacity (mls per second) are derived. The test is reliable and valid for a HNC population, with evidence to suggest patients can reliably self-assess [27–29]. The WST has been used as part of a battery of outcomes for swallowing interventions and different HNC treatments [8, 11, 30]. A minimally important difference for swallow capacity is estimated as being 4 ml/s [31].

2. Instrumental tests

Instrumental assessments such as videofluoroscopy swallow studies (VFSS) and fibreoptic endoscopic swallowing studies (FEES) directly observe swallowing function and numerous scales are available to record and interpret findings. Other instrumentation can measure discrete components of swallowing such as tongue strength and pharyngeal pressure generation during swallowing.

As a minimum, studies using VFSS or FEES usually report on swallow safety, i.e. the presence, depth and response to drink or food entering the airway. The Penetration-aspiration scale is a commonly used tool to capture this information, on an eight-point scale [32]. Reporting of the scale has been critiqued for its statistical interpretation [33]; however, it is often used as a primary outcome for swallowing rehabilitation trials in HNC [34, 35].

One of the criticisms of the Penetration-aspiration scale is that it fails to indicate the amount or frequency of aspiration — a key consideration in evaluating swallow safety. A recently developed validated scale, DIGEST-VF (Dynamic Imaging Grade of Swallowing Toxicity), addresses this flaw by grading the incidence, amount and frequency of penetration/aspiration (safety) and combining this information with a grading of pharyngeal residue (efficiency) to derive an overall score [36]. The authors advise that a standard VFSS protocol with a minimum of five thin liquid bolus trials, one pudding and one solid bolus is used when applying DIGEST-VF. DIGEST-VF is an outcome measure for a number of clinical trials [8, 11, 37]. Work had expanded to include a reliable and valid DIGEST scale for FEES [38]. Although both scales provide a single overall swallowing impairment score, they do not attempt to describe the underlying pathophysiology. This gap is addressed by the Modified Barium Swallow Impairment Profile (MBSImp) [39], a comprehensive standardized system of swallowing physiology, observed by VFSS. Preliminary guidance on a meaningful important difference for MBSImp has been published [40].

## HNC Treatment Modification to Reduce Dysphagia

### OPSCC

Over the past decade, the rise in HPV-positive oropharyngeal squamous cell cancer (OPSCC) has led to a number of multi-centre RCTs, addressing post-treatment dysphagia in this sub-group. Patients with this disease are typically younger at diagnosis and have good survival outcomes, meaning they could be living with treatment side effects for a long period. These patients may be offered minimally invasive surgery (MIS), using transoral robotic or laser techniques. Although dysphagia is common in the early post-operative period [41, 42], by 1 year, patients report similar swallowing function to that at baseline [43]. Alternatively, patients may be offered radiotherapy, with or without chemotherapy ((C)RT). ORATOR is a RCT comparing primary MIS with adjuvant (C)RT as required or primary (C)RT, with the MDADI as its primary outcome [9]. At 1 year, scores were better in the (C)RT group, but there was no meaningful important difference (10 points) between the two treatments. Findings suggest both treatments offer good swallowing-specific quality of life. ‘Best of’ is a European trial in recruitment, also comparing MDADI scores as its primary outcome for patients with early-stage OPSCC, supraglottic or hypopharyngeal cancer treated either with IMRT or MIS [10]. Finally, PATHOS is an international RCT comparing the de-intensification of adjuvant (C)RT) treatment following MIS for HPV-positive OPSCC, using the same co-primary endpoint [11].

### Advanced HNC

For advanced disease, treatment options typically include open surgery with reconstruction and adjuvant (C)RT, or primary non-surgical treatment. For open surgery, the degree of swallowing impairment will be influenced by numerous variables such as volume of tissue resected, type of closure and reconstruction and presence of a tracheostomy [44]. Primary non-surgical treatment techniques have developed to reduce radiation to tissues considered critical to swallowing. Single-arm studies of this technique suggest improved swallowing function [5]. DARS, an RCT has shown better patient-reported outcomes and improved diet scores for dysphagia-optimised IMRT compared to standard IMRT [8].

## Speech and Language Pathology Interventions

### Prehabilitation

The majority of prophylactic treatment paradigms encompass broad protocols which typically aim to strengthen and improve the range of movement of oropharyngeal musculature, countering the effects of post-treatment fibrosis, muscular atrophy and cranial neuropathies [45]. Prophylactic swallowing exercises have largely been embedded in clinical practice in many international cancer centres; however, despite a proliferation of trials, the potential benefit of implementing these exercises prior to and during cancer treatment remains equivocal [46–49]. Variability in the findings may be attributed to poor patient adherence, varying treatment protocols, lack of standardisation of outcome measures and low-quality studies [46, 50].

### Adherence

Several studies have emerged recently which attempt to address these limitations [13, 37, 51, 52, 54], largely focusing on promoting patient adherence to interventions, with some evidence indicating that increased adherence positively influences outcomes [53, 54]. Adherence-promoting interventions include behavioural change techniques [13], home-based mobile applications [51, 52] and virtual coaching [37]. A trial currently being undertaken aims to integrate behavioural change techniques into the clinical pathway to enhance engagement. Behavioural change is operationalised through education, individually tailored swallow exercise programme, goal-setting, self-monitoring and behavioural practice [13]. The clinician may also play a critical role in fostering adherence with a clinician-directed protocol producing significantly better adherence rates than patient-directed protocols [55]. However, even with good adherence to exercise protocols, uncertainty remains over treatment efficacy [37, 53, 54].

### Treatment Timing

Timing of interventions may also account for the variability in the current findings. Early intervention may have the potential to improve response rate to therapy for diet and quality of life [56]; however, the optimal timing of interventions within this timeframe is yet to be established. A large multi-centre RCT (*n* = 942) will attempt to elucidate this issue by determining the effectiveness of therapy provided at the point of dysphagia diagnosis in contrast to therapy delivered proactively [26•]. Alongside this, the trialists aim to ascertain whether intensity (low/high) and proactive maintenance of oral diet impact on swallow function. While proactive maintenance of oral intake is well established in clinical practice and represents a strong determinant for long-term swallow function [45, 57], its application alongside proactive exercise may offer synergistic effects.

### Personalised Therapy

Little is known about which treatment regimen or exercise is most effective and how tumour location/size and treatment toxicities interact to variably influence treatment effectiveness. In clinical practice, information about tumour site, radiotherapy course type, current swallow function and psychosocial functioning is utilised to individually tailor therapy in clinical practice [58]. However, dosimeter information is rarely used to direct dysphagia management [59] and may be important to guide future clinical protocols. New studies have emerged which focus on personalising interventions based on motivation, mental reserve, treatment toxicity and tumour site [13, 60]. Individually tailored therapy programmes combine functional skills training with progressive resistance training based on clinical examination of swallowing function [61]. Moving away from a one-size-fits-all model may help to reduce exercise and mental burden, since intensive loading will not be applicable to all.

### Intensity

High-intensity ‘bootcamp’ treatment programmes, based on the McNeil Dysphagia Therapy Programme [24], have emerged over the last few years [62, 63]. High-intensity activity overloads the system by activating residual muscles beyond their usual capacity which can lead to neuromuscular adaption [61]. Mass practice of functional swallows multiple times daily is advocated within these programmes, over a period of 3–10 weeks [62, 63]. These individualised therapy programmes place progressive load on the oropharyngeal musculature by varying fluid viscosity and solid texture which are prescribed in line with clinical and physiological data. Preliminary findings suggest certain patients are more responsive to intensive exercise regimens, due to variants of tumour size/location and treatment effects [63]. However, given that adherence rates to swallowing protocols are low [50], increasing treatment intensity is likely to be challenging in the clinical and research context.

### Future Directions

Expiratory Strength Muscle Training (EMST) utilises a device-driven exercise protocol which offers an alternative treatment paradigm. EMST targets (1) subglottic expiratory generating forces, allowing material to be expelled from the airway more efficiently through cough elicitation, and (2) submental suprahyoid activation with the aim of improving airway closure [64–67]. Both maximum expiratory pressure and swallow safety were found to improve significantly, following an 8-week programme of EMST [68]. While caution should be drawn at this stage since evidence is restricted to a small retrospective case series, five RCTs are in progress which should provide more definitive evidence in relation to treatment efficacy, allowing for potential clinical application [69–73].

Focusing on enhancing swallow function in isolation risks overlooking a fundamental process, the coordination of respiration with swallowing. A recent study has highlighted the potential benefits of training optimal respiratory-swallow coordination (swallowing during a pause in the mid to low tidal volumes of the expiratory cycle) using biofeedback in patients with HNC and chronic dysphagia [74]. Martin-Harris and colleagues found that respiratory-swallow training, of only 4 to 8 1-h sessions, in this population significantly improves swallow pathophysiology (laryngeal vestibule closure, tongue base retraction, bolus clearance) and airway protection [74]. Although these innovations are still in their infancy, they represent a move to considering systems rather than isolated functions and offer potential for further development and clinical adaptation (with instrumentation modification), as new evidence accumulates.

Manual therapies, targeting swallow musculature through passive and active stretching and soft muscle mobilisation, may offer a potential treatment to mitigate some of the long-term effects associated with radiation-induced fibrosis. In the USA, there is evidence of clinical application despite only speculative evidence for its effectiveness with patients with HNC [75]. Preliminary findings suggest manual therapies, often reserved as a reactive intervention for lymphedema, may in fact be tolerable and actually reduce localised pain during radiotherapy in this population [76]. In contrast, the MANTLE trial, currently in progress, utilises manual therapy reactively with the aim of improving cervical posture to fully optimise the swallow environment [77].

Dysphagia research activity has largely focused on an impairment-based approach, adhering closely to a biomedical model, and as such has largely neglected to address the wider psychosocial sequelae associated with dysphagia. However, recent research has sought to bridge this gap by combining cognitive behavioural therapy and behavioural swallow interventions (CB-EST) through individually tailored programmes [12]. Although effectiveness of the intervention is yet to be established, the principles of the intervention align with patient priorities identified in qualitative findings [78]. Future interventions need to consider the multifactorial nature of dysphagia and in doing so incorporate nutritional counselling and psychosocial functioning alongside physiological swallow function to meet the patient’s holistic needs.

### Technological Advances

The Covid-19 pandemic saw an explosion in the clinical adaptation of virtual platforms and applications, allowing patients to receive support from their own home. As we move to a model of hybrid working, applications, such as HNC virtual coach and Mobili-T, represent an adjunct, rather than a replacement, to standard care [37, 52]. The HNC virtual coach offers a holistic model of care, embodying swallowing exercises, education, social networking and nutritional counselling. Favourable outcomes in adherence rates and quality of life have been reported [37], highlighting its potential utility within a clinical setting.

Biofeedback devices such as ultrasound [79], manometry and sEMG [52, 74] offer the potential for patients to visualise the swallow structures engaged in real-time swallow-related activities. These augmentative tools have the potential to facilitate error identification and re-learning; however, further investigation is warranted given the costs of equipment prior to clinical adaptation. With advances in technology, such as the capacity to analyse large datasets, computational modelling and artificial intelligence [80], it will be possible gain an understanding of the impact of disease and treatment on swallow pathophysiology and thereby target highly individualised training programmes, based on these unique characteristics.

## Conclusion

Over the past decade, there has been a major increase in the evidence base to prevent or reduce severity of HNC dysphagia. Much of this has been achieved through collaborative, multi-centre research groups. Alongside this, our understanding of outcome measures has evolved, and importantly how to evaluate a meaningful change in outcome from the patient’s perspective. By harmonising these outcomes, meta-analysis becomes a possibility for the future.

Much of the focus of either de-intensifying or modifying HNC treatment has been on early-stage OPSCC. Further work is needed to look at other sub-groups and their resultant functional deficits. For SLP interventions, ambiguity over the extent to which exercise is preventative and/or ameliorative remains. We suspect integration of device-driven, functional skills–based activity and strengthening exercises, which are individually tailored based on tumour site and dosimeter data may yield the best results within a holistic model of care which addresses psychosocial and nutritional counselling.
